# Epidemiology and clinical features of movement disorders in a Mexican Mestizo cohort: a 10-year tertiary-center study

**DOI:** 10.1055/s-0046-1829047

**Published:** 2026-10-09

**Authors:** Katia Carmina Farias-Moreno, Leonel Villa-Villegas, Biagio David González-Ruffino, Luz Gabriela Lira-Jaime, Karla Berenice Carrazco-Peña, Enrique Gómez-Figueroa, Carlos Zúñiga-Ramírez

**Affiliations:** 1 Antiguo Hospital Civil de Guadalajara Fray Antonio Alcalde, Movement Disorders and Neurodegenerative Diseases Unit, Guadalajara, Jalisco, México.; 2 Universidad de Colima, Faculty of Medicine, Colima, México.; 3 Antiguo Hospital Civil de Guadalajara Fray Antonio Alcalde, Department of Neurology, Guadalajara, Jalisco, México.

**Keywords:** Movement Disorders, Parkinson Disease, Epidemiology, Mexico, Tremor, Cohort Studies

## Abstract

**Background:**

Movement disorders comprise a heterogeneous spectrum of neurological conditions characterized by impaired motor control. Limited epidemiological data exist for Latin American populations, particularly for Mexican Mestizos.

**Objective:**

To characterize the epidemiological profile and clinical features of movement disorders in a large cohort of Mexican Mestizo patients evaluated at a public tertiary referral center in western Mexico.

**Methods:**

We conducted a 10-year retrospective cohort study (2012–2022) analyzing medical records from the Movement Disorders and Neurodegenerative Diseases Unit at the Hospital Civil de Guadalajara, a public tertiary university hospital from the western region of Mexico. Inclusion criteria were confirmed movement disorder diagnosis and a minimum of two clinical evaluations. Binary logistic regression was used to calculate odds ratios (OR) and 95% confidence intervals (CIs).

**Results:**

Among 1,830 records (mean age 58.5 ± 17.0 years, 53.9% female, all self-identified as Mexican Mestizo), hypokinetic disorders comprised 63.9% (n = 1,170) and hyperkinetic disorders 36.1% (n = 660). Parkinsonism (63.6%, n = 1,163) and tremor (11.3%, n = 206) were the most frequent subtypes. Hypokinetic disorders were associated with older age (64.2 ± 12.8 vs. 48.5 ± 18.9 years, *p* < 0.001), hypertension (OR = 2.29; 1.78–2.95; *p* = 0.001), and smoking (OR = 1.51; 1.13–2.03; *p* = 0.005). Hyperkinetic disorders were associated with male sex (OR = 1.47; 1.19–1.82; *p* < 0.001), family history (OR = 1.92; 1.49–2.47; *p* < 0.001), and functional presentations (OR = 11.0; 5.53–22.14; *p* < 0.001).

**Conclusion:**

This is the largest reported Mexican Mestizo cohort with movement disorders evaluated in a single public tertiary center. Findings reveal distinct demographic and clinical patterns and may inform integrated cardiovascular–neurological care models, screening protocols, and resource allocation within the Mexican public health system.

## INTRODUCTION

Movement disorders constitute a heterogeneous group of neurological conditions characterized by impaired motor control resulting from basal ganglia–thalamocortical circuit dysfunction. 1 These disorders are classified into hypokinetic categories (characterized by bradykinesia, rigidity, and postural instability) and hyperkinetic categories (including dystonia, chorea, tremor, and tics), based on underlying pathophysiological mechanisms. 2 3 Contemporary understanding reveals that these pathological states involve not only changes in firing rate but also abnormal oscillatory patterns that disrupt normal neural communication. 4

Global epidemiological studies reveal significant regional disparities in movement disorder prevalence, ranging from 49 per 100,000 in Sub-Saharan Africa to 1,081 per 100,000 in Latin America and the Caribbean. 5 The Global Burden of Disease study projects that Parkinson's disease cases will reach 25.2 million by 2050, marking a 112% increase from 2021. 6 Sex disparities are well documented, with Parkinson's disease showing male predominance (overall ratio 1.18), while functional movement disorders predominantly affect females. 7 8

In Latin America, high-quality epidemiological data remains scarce. A multicenter study reported a parkinsonism prevalence of 8.0%, with a 95% confidence interval (CI) of 7.6–8.5%, among adults aged ≥ 65 years. 9 However, Latin America is genetically and clinically heterogeneous: present-day Mexicans are the result of admixture between Spaniards and Amerindians, with a smaller African contribution, and Mexican Mestizos display a regional gradient in genetic ancestry that differs from the Andean, Southern Cone, and Caribbean populations. 10 Most epidemiological insights derive from high-income countries, creating critical knowledge gaps for clinical practice in admixed Mexican Mestizo populations, whose genetic background, comorbidity profile, and healthcare system differ markedly from those of other Latin American countries.

Understanding regional patterns enables evidence-based clinical decision-making and informs specialized center development. This study aims to characterize the epidemiological and clinical profile of movement disorders in the largest Mexican Mestizo cohort reported to date from a single public tertiary center, providing actionable insights for clinical practice and healthcare planning in Mexico, while explicitly acknowledging the limits to extrapolation to other Latin American populations.

## METHODS

### Study design and setting

We conducted a retrospective cohort study at the Movement Disorders and Neurodegenerative Diseases Unit, Hospital Civil de Guadalajara Fray Antonio Alcalde (HCG-FAA), reviewing medical records from January 2012 to December 2022.

### Hospital catchment area, access mechanisms, and comparability with the Mexican population

The HCG-FAA is a public, decentralized, tertiary, teaching hospital affiliated with the University of Guadalajara and is part of the Jalisco State Health System. It is the largest public health institution in western Mexico, with more than 1,150,000 outpatient visits per year for the entire complex. Access to the Movement Disorders Unit occurs through three pathways:

- Direct ambulatory referral from primary or secondary care facilities of the State Health System and from community physicians for patients without social security;
- Internal transfer from emergency or general neurology services within HCG-FAA when a movement disorder is identified; and
- Self-referral by patients seeking specialized care.

The patient population served by HCG-FAA is predominantly Mexican Mestizo. Genetic studies have shown that Mexicans from Jalisco and the western region exhibit a higher European admixture component than Mestizos from central or southeastern Mexico. 10

### Participant selection

The inclusion criteria were a confirmed movement disorder diagnosis according to established clinical criteria; a minimum of two clinical evaluations to establish disease course; complete demographic and clinical data; and age ≥ 18 years at the first evaluation.

Exclusion criteria were unclear or unconfirmed diagnosis, incomplete data, or secondary movement disorders without an established primary diagnosis. Consecutive sampling was used, including all subjects who sequentially met the eligibility criteria, which minimized selection bias within the referral population and ensured representativeness of the tertiary-center catchment. Figure 1 presents the study flowchart, detailing patient identification, screening, reasons for exclusion, and the final cohort included according to Strengthening the Reporting of Observational studies in Epidemiology (STROBE) recommendations.

**Figure 1:**
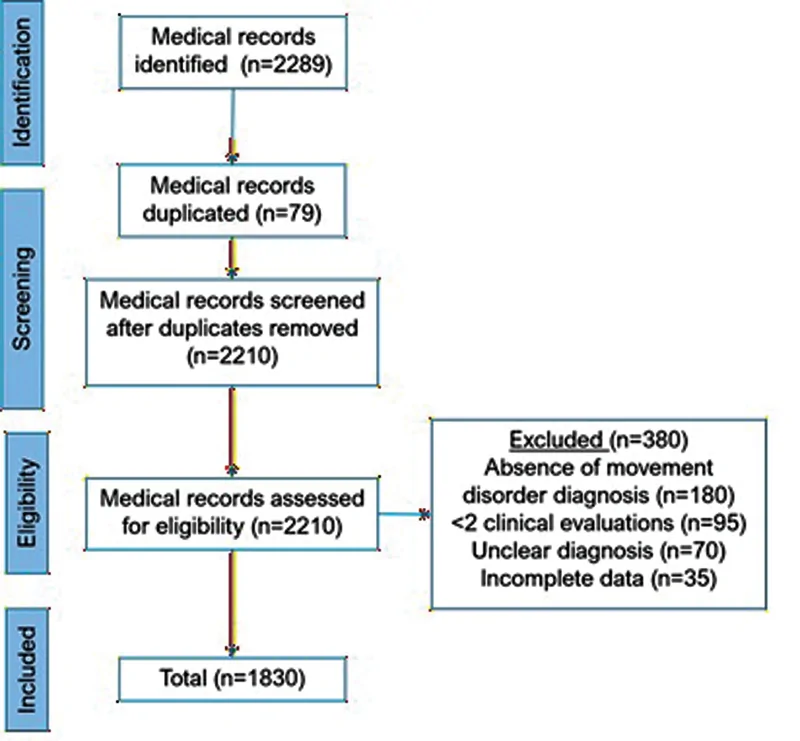
Study flowchart, providing an overview of the subject selection according to Strengthening the Reporting of Observational studies in Epidemiology (STROBE) guidelines. Missing data were handled using complete case analysis. Subjects with incomplete key variables were excluded from specific analyses.

### Variables and measurements

Collected variables included demographics (age, sex, self-reported ethnicity, and follow-up duration), family history across two generations, and comorbidities (hypertension, type 2 diabetes, dyslipidemia, and cardiovascular disease). Substance use included smoking status, alcohol consumption, and history of illicit drug use. Movement disorders were classified as hypo- or hyperkinetic, with the following subtypes: parkinsonism, tremor, dystonia, chorea, myoclonus, tics, ataxia, rapid eye movement (REM) sleep behavior disorder (SBD), and specialized categories (genetic, functional, and atypical parkinsonism).

All diagnoses were assigned by board-certified neurologists specializing in movement disorders using established diagnostic criteria. Functional movement disorders (FMD) were diagnosed using the laboratory-supported criteria. 11 The genetic nature of a movement disorder was defined a priori by the presence of at least one of the following:

- a confirmed pathogenic or likely pathogenic variant identified through targeted molecular genetic testing (single-gene Sanger sequencing or next-generation sequencing panels),
- a clinically established hereditary syndrome (e.g., Huntington's disease, hereditary spinocerebellar ataxia, or dystonia-parkinsonism syndromes) supported by a positive multigenerational family history compatible with Mendelian inheritance and a consistent phenotype, when molecular testing was unavailable; or
- both criteria combined.

### Statistical analysis

Descriptive statistics were calculated for all variables. Normality was assessed using the Kolmogorov–Smirnov test with Lilliefors correction. Student's *t* -test and analysis of variance (ANOVA) were used to analyze normally distributed continuous variables; the Mann-Whitney U test was used for non-normally distributed variables; and the chi-square test was used for categorical variables. Binary logistic regression with stepwise forward selection was used to calculate odds ratios (OR) with 95% CI. Model fit was assessed using the Hosmer-Lemeshow test, and discriminative ability was evaluated using receiver operating characteristic (ROC) curve analysis. Statistical significance was set at *p* < 0.05. Analyses were performed using IBM SPSS Statistics for Windows (IBM Corp.), version 29.0, and R (R Foundation for Statistical Computing), version 4.4.

### Ethical considerations

Ethics approval was obtained from the Institutional Review Board and Bioethics Committee of Hospital Civil de Guadalajara (Protocol #HCG/FAACEI-142/25, January 2025). Informed consent was waived because the study employed a retrospective design, using deidentified existing medical records, involving no investigational procedures or interventions, presenting minimal risk to participants. Furthermore, obtaining consent was not feasible given the historical cohort, spanning 2012 through 2022. Data deidentification was completed using the HIPAA Safe Harbor Method, removing all 18 identifiers prior to analysis. The present study was conducted in compliance with the Declaration of Helsinki and Mexican regulations for research involving human subjects.

## RESULTS

### Demographic characteristics

Among 1,830 subjects evaluated, the mean age was 58.5 ± 17.0 years (range: 4–92 years), with female predominance (53.9%; n = 987) and a mean follow-up duration of 19.5 ± 24.3 months. All subjects were Mexican-born and self-identified as Mexican Mestizo ( Table 1 ).

**Table 1:** Sample characteristics

Epidemiological characteristics	n (%) or mean (SD)
Age (years), mean (SD)	58.5 (17.0)
1–25	123 (6.8)
26–50	359 (19.6)
51–70	1,080 (59.0)
≥ 71	268 (14.6)
Female sex	987 (53.9)
Mexican Mestizo (self-identified)	1,830 (100)
Family history (present)	360 (19.7)
**Substance use**	
Smoking	379 (20.7)
Alcohol use	448 (24.5)
Drug abuse	64 (3.5)
**Comorbidities**	
Hypertension	501 (27.4)
Type 2 diabetes	326 (17.8)
Dyslipidemia	364 (19.9)
**Primary diagnoses**	
Parkinsonism	714 (39.0)
Parkinson's disease	381 (20.8)
Essential tremor	192 (10.5)
Huntington's disease	79 (4.3)
Ataxia	68 (3.7)
Facial spasm	47 (2.6)
**Movement disorder classification**	
Hypokinetic	1,170 (63.9)
Hyperkinetic	660 (36.1)
**Movement disorder subtypes**	
Parkinsonism	1,163 (63.6)
Tremor	206 (11.3)
Dystonia	173 (9.5)
Chorea	103 (5.6)
Ataxia	84 (4.6)
Myoclonus	57 (3.1)
Tics	12 (0.7)
Stereotypies	11 (0.6)
REM sleep behavior disorder	9 (0.5)
**Movement disorder features**	
Atypical parkinsonism	60 (3.3)
Genetic origin	95 (5.2)
Functional movement disorder	67 (3.7)

Abbreviations: REM, rapid eye movement; SD, standard deviation. Note: Data presented as n (%) or mean (SD).

### Movement disorder classification

Hypokinetic disorders comprised 63.9% (n = 1,170) of cases, while hyperkinetic disorders accounted for 36.1% (n = 660). Parkinsonism (63.6%; n = 1,163), tremor (11.3%; n = 206), and dystonia (9.5%; n = 173) were the most frequent subtypes. Primary diagnoses included parkinsonism (39.0%; n = 714), Parkinson's disease (20.8%; n = 381), and essential tremor (10.5%; n = 192).

Within the hypokinetic group (n = 1,170), the great majority were classified as parkinsonism in its broad sense (n = 1,163), including Parkinson's disease, atypical parkinsonism, and parkinsonism. The seven non-Parkinsonian hypokinetic cases corresponded to bradykinetic-rigid syndromes that did not fulfill diagnostic criteria for parkinsonism, predominantly comprising drug-induced bradykinesia in the context of antidopaminergic exposure (n = 4) and progressive bradykinesia within complex syndromes, such as corticobasal-like presentations not meeting full diagnostic criteria for corticobasal syndrome and isolated muscular rigidity/stiffness syndromes (n = 3). This explanation reconciles the previously observed numerical difference between hypokinetic disorders (n = 1,170) and parkinsonism (n = 1,163).

### Age and sex patterns

Hypokinetic disorders were associated with significantly older age (64.2 ± 12.8 years) compared with hyperkinetic disorders (48.5 ± 18.9 years; *p* < 0.001). Males predominated in hypokinetic disorders (50.1%; *n* = 587), while females were more frequent in hyperkinetic disorders (60.6%, *n* = 400; *p* < 0.001). Detailed age and sex stratification and comparative analysis across movement disorder subtypes are presented in Table 2 .

**Table 2:** Mean age (years) by movement disorder type, stratified by comorbidities and risk factors

Variable	Hypokinetic	Hyperkinetic	*p* -value
Family history	59.3 ± 16.7	55.5 ± 17.7	< 0.001*
Type 2 diabetes	57.1 ± 17.7	65.2 ± 11.5	< 0.001*
Hypertension	55.4 ± 18.1	62.0 ± 13.6	< 0.001*
Dyslipidemia	56.8 ± 17.9	65.5 ± 10.5	< 0.001*
Smoking	57.9 ± 7.6	60.9 ± 14.3	0.003*
Alcohol use	58.3 ± 17.6	59.3 ± 15.2	0.20
Drug abuse	59.0 ± 17.0	46.6 ± 13.6	< 0.001*
Genetic origin	59.2 ± 16.8	46.4 ± 16.0	< 0.001*
Functional movement disorder	59.3 ± 16.7	39.7 ± 14.1	< 0.001*

Notes: Data presented as mean ± SD. *Statistically significant. The *p* -values were calculated using Student's *t* -test for independent samples.

Figure 2 illustrates the age distribution across subtypes, revealing distinct patterns. Parkinsonism showed the highest mean age (64.4 ± 12.5 years; *n* = 1,163), while stereotypies showed the youngest mean age (24.5 ± 8.9 years, *n* = 11). Age-stratified analysis ( Table 3 ) confirmed the predominance of parkinsonism and tremor in subjects ≥ 50 years, while dystonia, tics, and stereotypies clustered in subjects < 25 years.

**Table 3:** Age distribution by movement disorder subtypes

Subtype (n)	1–25 years	26–50 years	51–70 years	> 71 years
Parkinsonism (n = 1,163)	14 (1.2%)	130 (11.2%)	800 (68.8%)	219 (18.8%)
Tremor (n = 206)	16 (7.8%)	43 (20.9%)	123 (59.7%)	24 (11.7%)
Dystonia (n = 173)	54 (31.2%)	64 (37.0%)	44 (25.4%)	11 (6.4%)
Chorea (n = 103)	6 (5.8%)	49 (47.6%)	40 (38.8%)	8 (7.8%)
Ataxia (n = 84)	11 (13.1%)	40 (47.6%)	30 (35.7%)	3 (3.6%)
Myoclonus (n = 57)	4 (7.0%)	16 (28.1%)	34 (59.6%)	3 (5.3%)
Tics (n = 12)	6 (50.0%)	6 (50.0%)	—	—
Stereotypies (n = 11)	8 (72.7%)	3 (27.3%)	—	—
REM sleep behavior disorder (n = 9)	1 (11.1%)	2 (22.2%)	6 (66.7%)	—
Gait disorder (n = 4)	2 (50.0%)	1 (25.0%)	1 (25.0%)	—
Muscular stiffness (n = 3)	1 (33.3%)	2 (66.7%)	—	—
Athetosis (n = 3)	—	1 (33.3%)	2 (66.7%)	—
Akathisia (n = 2)	—	2 (100%)	—	—

Notes: Data presented as n (%). Calculated using proportions; Chi-squared test with Yates' correction ( *p* < 0.001).

**Figure 2:**
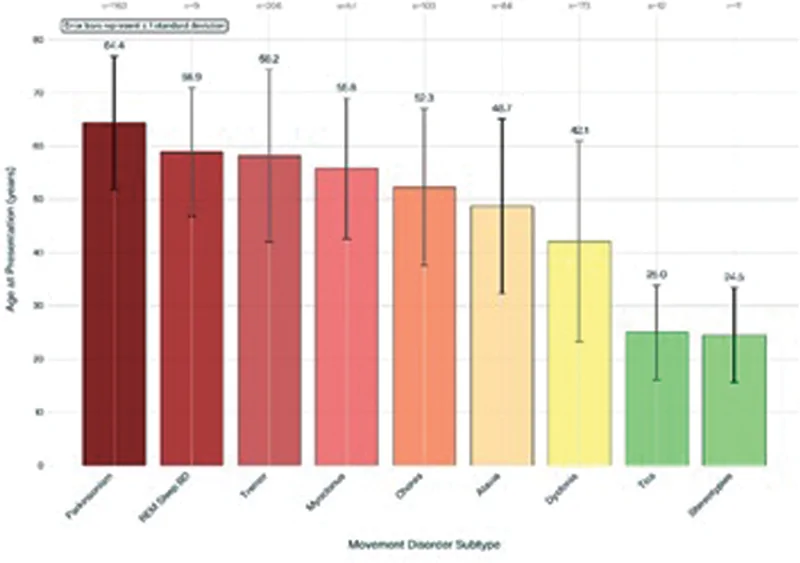
Age patterns by movement disorder subtype. Dystonia and tics are primarily encountered in pediatric and young adult populations, whereas ataxia, parkinsonism, and tremor predominantly affect older adults. **Abbreviation:** REM sleep BD, rapid eye movement sleep behavior disorder.

### Clinical associations

Family history of movement disorders was reported in 19.7% (n = 360) of subjects. Hypertension (27.4%; n = 501) and type 2 diabetes (17.8%; n = 326) were the most common comorbidities. Parkinsonism was associated with male sex (OR = 1.5; 1.2–1.8, *p* < 0.001), particularly in Parkinson's disease (OR = 1.4; 1.1–1.8, *p* = 0.001). Conversely, tremor was associated with female sex (OR = 2.0; 1.4–2.7, *p* < 0.001), primarily for essential tremor (OR = 1.6; 1.2–2.4, *p* = 0.009).

### Specialized categories

A genetic etiology was confirmed in 5.2% ( *n* = 95) of cases, with parkinsonism (59.0%; *n* = 56), ataxias (33.6%; *n* = 32), and dystonia (7.4%; *n* = 7) being the most frequent. Functional movement disorders comprised 3.7% ( *n* = 67), predominantly presenting as dystonia (70.1%; *n* = 47), parkinsonism (9.0%; *n* = 6), and tremor (7.5%; *n* = 5). Atypical parkinsonism represented 3.3% ( *n* = 60), comprising progressive supranuclear palsy (55.0%; *n* = 33), multiple system atrophy (23.4%; *n* = 14), and corticobasal syndrome (21.6%; *n* = 13).

### Multivariate analysis

Binary logistic regression ( Table 4 ) identified significant predictors after confirming model calibration (Hosmer-Lemeshow *p* = 0.093) and absence of multicollinearity, with a variance inflation factor (VIF) < 1.25.

**Table 4:** Regression coefficients and odds ratios for hypokinetic and hyperkinetic movement disorders

Variable	B (SE)	Hypokinetic OR (95% CI)	Hyperkinetic OR (95% CI)	*p* -value
Male sex	-0.38 (0.11)	0.67 (0.55–0.83)	1.47 (1.19–1.82)	< 0.001
Family history	-0.65 (0.13)	0.52 (0.40–0.66)	1.92 (1.49–2.47)	< 0.001
Hypertension	0.83 (0.15)	2.29 (1.78–2.95)	0.43 (0.33–0.56)	0.001
Smoking	0.41 (0.18)	1.51 (1.13–2.03)	0.65 (0.49–0.88)	0.005
Drug abuse	-0.65 (0.29)	0.51 (0.29–0.92)	1.92 (1.08–3.41)	0.02
Functional movement disorder	-2.40 (0.35)	0.09 (0.04–0.18)	11.0 (5.53–22.14)	< 0.001

Abbreviations: B, regression coefficient; CI, confidence interval; OR, odds ratio; SE, standard error; VIF, variance inflation factor. Notes: Adjusted binary logistic regression model: Hosmer-Lemeshow *p* = 0.09; Cox-Snell R ^2^ = 0.091; Nagelkerke R ^2^ = 0.120; VIF <1.2; standardized residuals mean = 0.0002, SD = 1.0; no residuals exceeded ± 3.0; Cook's mean distance = 0.0039; leverage < 0.2.

For hyperkinetic disorders: male sex (OR = 1.47; 95% CI: 1.19–1.82; *p* < 0.001), family history (OR = 1.92; 95% CI: 1.49–2.47; *p* < 0.001), illicit drug use (OR = 1.92; 95% CI: 1.08–3.41; *p* = 0.02), and functional movement disorders (OR = 11.0; 95% CI: 5.53–22.14; *p* < 0.001). For hypokinetic disorders: hypertension (OR = 2.29; 95% CI: 1.78–2.95; *p* = 0.001) and smoking (OR = 1.51; 95% CI: 1.13–2.03; *p* = 0.005). The ROC analysis yielded an area under the curve (AUC) of 0.657 (95% CI: 0.631–0.682; *p* < 0.001), as shown in Figure 3 .

**Figure 3:**
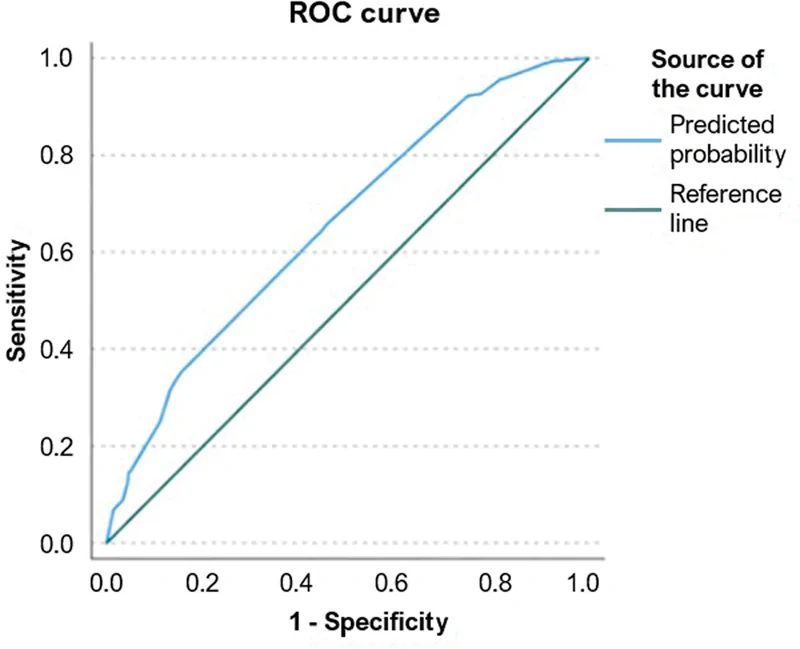
The ROC curve for the logistic regression model, illustrating the versus specificity for predicted probabilities in the cohort. The AUC was 0.657 (95% CI: 0.631–0.682; *p* < 0.001), indicating statistically significant discriminative ability. At the optimal cut-off determined by the Youden Index (predicted probability = 0.558), the model yielded a sensitivity of 92.2% and a specificity of 25.2%. **Abbreviations:** AUC, area under the curve; ROC, receiver operating characteristic.

## DISCUSSION

### Clinical implications and practice relevance for the Mexican Mestizo population

This comprehensive analysis of the largest reported single-center cohort of Mexican Mestizo patients with movement disorders provides actionable insights for clinical practice and healthcare planning at our institution and within the Mexican public health system. The predominance of hypokinetic disorders (63.9%) with distinct demographic patterns directly informs evidence-based screening protocols and resource allocation strategies for the uninsured population served by the Jalisco State Health System. Because our population is genetically, clinically, and administratively distinct from cohorts reported elsewhere in Latin America, we deliberately frame these implications for the Mexican Mestizo context first, and only secondarily for other Latin American settings that share comparable admixture and healthcare characteristics.

### Local tertiary-care referral patterns and the Mexican healthcare system

A unique facet of the present cohort is its origin within the Mexican public, tertiary, university-based healthcare model: HCG-FAA receives patients from a variety of backgrounds in western Mexico, mostly without social security. This context likely explains the high diagnostic complexity of our cohort, the relatively advanced disease stages observed at first evaluation, and the substantial proportion of atypical parkinsonism (3.3%). It also implies that our results are particularly relevant for designing referral pathways, training programs, and resource allocation within Mexico's public hospital network. Therefore, healthcare-system implications, including the need for dedicated movement-disorder fellowships, are the central messages of this study.

### Age and sex screening implications

The significant age differential between hypokinetic (64.2 ± 12.8 years) and hyperkinetic disorders (48.5 ± 18.9 years) supports age-stratified screening approaches in our setting. For clinical practice, this suggests prioritizing parkinsonism evaluation in males > 50 years while maintaining heightened awareness for hyperkinetic disorders in younger females. The strong male association with parkinsonism (OR = 1.5; *p* < 0.001) and the female predominance in tremor disorders (OR = 2.0; *p* < 0.001) provide evidence-based guidance for sex-specific clinical assessments in Mexican Mestizo patients. 12

### Cardiovascular integration in clinical care

The striking association between hypertension and hypokinetic disorders (OR = 2.29; *p* = 0.001) has clinical relevance for integrated care models in Mexican Mestizo patients, in whom cardiometabolic comorbidity is highly prevalent. This finding suggests that cardiovascular screening should be prioritized in patients with parkinsonism, while movement disorder evaluation may be warranted in hypertensive patients presenting with subtle motor symptoms. The association with smoking (OR = 1.51; *p* = 0.005) further supports comprehensive cardiovascular risk assessment in movement disorder clinics. 13 14 However, this association should be interpreted with caution, as it may partly reflect well-known biases inherent to retrospective tertiary-center data, including an age confounder (older patients have a higher prevalence of both hypertension and parkinsonism), surveillance bias (those with established hypertension undergo more medical evaluations, increasing likelihood of subtle parkinsonism being detected), and shared cardiovascular risk factors. Therefore, the OR of 2.29 should be regarded as hypothesis-generating rather than as direct evidence of a causal pathophysiological link.

These cardiovascular associations align with emerging evidence suggesting shared pathophysiological mechanisms. For clinical practice, this emphasizes the importance of multidisciplinary approaches incorporating cardiology consultation, particularly given potential drug interactions between cardiovascular medications and movement disorder therapies. 15 Public healthcare systems serving Mexican Mestizo populations should consider integrated care pathways combining movement disorder and cardiovascular expertise.

### Functional movement disorders: recognition and management

The identification of functional movement disorders in 3.7% of cases, with an exceptionally strong statistical association (OR = 11.0; *p* < 0.001), should be interpreted as evidence of underrecognition rather than as a reliable estimate of true prevalence. The predominance of dystonia presentations (70.1%) and the younger age profile provide specific clinical indicators that should prompt active screening. 16

For practice implementation, this suggests incorporating functional movement disorder assessment protocols in all movement disorder evaluations within our system, particularly for younger Mexican Mestizo patients presenting with dystonia-like symptoms. The high OR indicates that functional presentations may be more common than previously recognized. 17

## Study limitations

Several limitations warrant consideration. First, this is a single-center, tertiary-care, public referral hospital cohort, which probably overrepresents complex, atypical, and advanced cases while underrepresenting mild or common conditions usually managed in primary care (such as mild essential tremor or early restless legs syndrome). Second, the retrospective design limits the standardization of data collection and the granularity of clinical scales, and missing data in some variables may have affected estimates.

Third, although our cohort is the largest single-center Mexican Mestizo cohort reported to date, its demographic, ethnic, and healthcare-system features mean that the findings are most directly applicable to the Mexican population, and not necessarily to the entire Latin America, whose countries differ substantially in genetic ancestry, comorbidity profile, and health system architecture. Fourth, the modest discriminative capacity of the multivariate model (AUC = 0.657) suggests that additional unmeasured variables (e.g., environmental exposures, occupational history, and detailed genetic data) influence movement disorder patterns. Fifth, the retrospective ascertainment of functional movement disorders almost certainly underestimates their true frequency.

Despite these limitations, the large sample size (n = 1,830) and the 10-year time span provide robust estimates for the specialized public-care population in western Mexico and a useful benchmark for other national tertiary centers.

In conclusion, this is the largest single-center Mexican Mestizo cohort with movement disorders reported to date ( *n* = 1,830), and it reveals distinct patterns with clinical relevance for Mexico and, with appropriate caution, for other Latin American populations of comparable admixture and healthcare context. Hypokinetic disorders were predominant (63.9%), showing a male association (OR = 1.5; *p* < 0.001) and significantly older age (64.2 ± 12.8 years), while hyperkinetic disorders showed female predominance (tremor OR = 2.0; *p* < 0.001) and younger age (48.5 ± 18.9 years). The strong cardiovascular association, particularly hypertension with hypokinetic disorders (OR = 2.29; *p* = 0.001), supports integrated care models within the Mexican public health system, although shared age and surveillance biases should be considered.

These findings provide evidence to support sex- and age-stratified screening protocols, resource allocation, and treatment planning in the Mexican population. The identification of functional movement disorders (3.7%) and genetic etiologies (5.2%) emphasizes the need for specialized expertise, structured diagnostic protocols, and access to molecular testing in Mexican movement disorder centers.
